# Helpful or harmful? How cancer beliefs and information seeking relate to depression in U.S. adults

**DOI:** 10.1371/journal.pone.0346262

**Published:** 2026-04-02

**Authors:** Ama Gyesiwaa Quansah, Helena Baffoe, Solomon Eshun

**Affiliations:** 1 Department of Communication Studies, Texas Tech University, Lubbock, Texas, United States of America; 2 Department of Sociology, Anthropology, and Social Work, Texas Tech University, Lubbock, Texas, United States of America; 3 Department of Psychiatry, Texas Tech University Health Sciences Center, Lubbock, Texas, United States of America; 4 Department of Biostatistics, University of North Carolina at Chapel Hill, Chapel Hill, North Carolina, United States of America; The Hong Kong Polytechnic University, HONG KONG

## Abstract

This study examined how cancer-related beliefs, information-seeking behaviors, and discussions about health with family or friends relate to depressive symptoms (PHQ-2 ≥ 3) among U.S. adults, using data from the 2024 Health Information National Trends Survey (HINTS 7; unweighted N = 6,826). Associations were estimated using survey-weighted logistic regression with jackknife replicate weights, adjusting for sociodemographic factors and personal or family cancer history; results are reported as adjusted odds ratios (ORs) with 95% confidence intervals (CIs). Weighted estimates indicate that approximately 15.5% of respondents screened positive for depression. Fatalistic beliefs, particularly the views that everything causes cancer (OR = 1.86; 95% CI: 1.39–2.48), prevention is not possible (OR = 1.69; 95% CI: 1.25–2.28), and cancer automatically means death (OR = 1.75; 95% CI: 1.31–2.34), were significantly associated with higher odds of screening positive for depression. In contrast, neither cancer information seeking (OR = 1.12; 95% CI: 0.83–1.51) nor discussions about health with family or friends (OR = 0.90; 95% CI: 0.62–1.30) showed a significant independent association with depression. In moderation analyses, discussions about health with family or friends weakened the positive association between each fatalistic belief and depression, but these interaction effects were not statistically significant. Sensitivity analyses using multiple imputation for missing data and restricting the analyses to respondents without a personal cancer history produced consistent results. Theoretical and practical implications of these findings are discussed.

## Introduction

Cancer remains one of the leading causes of death in the United States [1,2] and occupies a prominent place in public discourse. Through media coverage, prevention campaigns, and personal stories shared in social circles, individuals are regularly exposed to information regarding cancer risk and survivorship. This cumulative exposure influences how the public perceive the disease, leading many to form strong impressions of what cancer means and, perhaps, how much control they might have over it [3,4]. Some may come to see cancer as something that can be prevented or treated, while others may perceive it as simply unavoidable.

These beliefs about controllability affect how people process cancer information. According to the Cognitive-Social Health Information Processing (C-SHIP) model, people do not interpret health threats in a vacuum; instead, they filter new information through their existing belief systems, which then dictate their emotional and behavioral reactions [5,6]. Within this framework, a belief that cancer cannot be controlled may evoke feelings of distress and helplessness [7]. One structured expression of this perceived lack of control is cancer fatalism, which includes the notion that everything causes cancer, that nothing can prevent it, or that a diagnosis inevitably leads to death [4,8,9].

Prior research shows that individuals who hold fatalistic beliefs about cancer are less likely to participate in screening or adopt preventive behaviors [4,7,9,10]. Some studies have also linked cancer fatalism to depression, though these investigations have primarily focused on college students or cancer survivors [11–13], leaving open the question of whether similar associations are present in the broader adult population. Fatalistic beliefs are not confined to individuals with a cancer diagnosis [4,14,15]. In fact, these beliefs can even form from seeing what others go through with cancer, which may foster feelings of helplessness, a core feature of depression [16]. As a result, it is reasonable to examine the emotional impact of fatalistic beliefs in the general adult population.

Aside beliefs, many people try to handle cancer concerns through communication. One common approach is information seeking. When cancer feels uncertain, people turn to online resources, news coverage, or health professionals to make sense of the situation. Uncertainty Management Theory (UMT) frames this behavior as an effort to reduce uncertainty by gaining understanding, reassurance, or a sense of control [17–21]. However, information seeking can sometimes backfire, leaving people more anxious if the information is frightening, conflicting, or unclear [10,19,22–25]. Another coping approach involves interpersonal health discussions with family and friends, which provide emotional and social support, and often promote understanding, hope and relief as individuals process concerns [26–28]. Despite these insights, it remains unclear how information seeking or health discussions with friends or family relate to depressive symptoms in the general adult population. It is also unknown whether discussions with friends or family can modify the relationship between fatalistic cancer beliefs and depression.

The present study examines the associations between cancer fatalism, cancer information seeking, interpersonal health discussions, and depressive symptoms in a nationally representative sample of U.S. adults. Consistent with the C-SHIP framework and prior research on cancer survivors, we hypothesize that endorsement of fatalistic cancer beliefs will be associated with higher odds of screening positive for depression (H1). Furthermore, we expect that the potential distress of encountering threatening information means that individuals seeking cancer-related information will have higher odds of depression (H2). In contrast, because interpersonal discussions can facilitate shared coping, we hypothesize that individuals who talk about their health with family or friends will have lower odds of screening positive for depression (H3). Finally, we hypothesize that talking about your health with family or friends will moderate the relationship between cancer fatalism and depression, such that the association will be weaker among individuals who engage in these conversations (H4).

## Methods

### Data source and information

This study analyzed data from the Health Information National Trends Survey (HINTS 7), a nationally representative, cross-sectional survey administered by the National Cancer Institute (NCI) in 2024. HINTS collects data from U.S. adults aged 18 years and older to monitor how they access, understand, and use information related to cancer and general health. The survey includes measures of health behaviors, beliefs, knowledge, and communication practices. Detailed information on survey design, sampling procedures, and questionnaire content is available on the HINTS website. The dataset included 7,278 respondents, encompassing those with and without a personal history of cancer. Because the outcome of interest (depressive symptoms) was measured using the Patient Health Questionnaire (PHQ-2), respondents missing either of the two PHQ-2 items could not be scored or classified. Therefore, those with missing PHQ-2 data were excluded, resulting in a final analytic sample of 6,826.

Since the data are publicly available and fully de-identified, ethical approval was not required.

### Measures

#### Dependent variable.

The dependent variable was depressive symptoms, measured using PHQ-2, a validated screening tool for major depressive disorder [29,30]. In HINTS 7, respondents reported how often over the past two weeks they experienced (1) little interest or pleasure in doing things and (2) feelings of sadness, depression, or hopelessness. The items were originally coded 1 (“Nearly every day”) to 4 (“Not at all”) in the dataset but were reverse-coded and rescaled (0 = “Not at all” to 3 = “Nearly every day”) following standard scoring conventions. The two items were summed (0–6), with scores of 3 or more indicating a positive depression screen. The PHQ-2 demonstrated acceptable internal consistency (*r* = 0.72; Cronbach’s α = 0.83).

#### Independent variables.

The main independent variables (IVs) were cancer fatalism, cancer information seeking, and interpersonal health discussions, each examined in a separate model. Cancer fatalism was measured using three HINTS 7 items: (B1) “It seems like everything causes cancer,” (B2) “There is not much you can do to lower your chances of getting cancer,” and (B3) “When I think about cancer, I automatically think about death.” Responses were orignally measured on a four-point Likert scale (1 = strongly disagree to 4 = strongly agree) and subsequently dichotomized as “agree” (agree or strongly agree) versus “disagree” (disagree or strongly disagree). The items were weakly correlated (B1–B2: *r* = 0.21; B1–B3: *r* = 0.25; B2–B3: *r* = 0.22; all *p* <0 .001) and had low internal consistency (Cronbach’s α = 0.46), so they were analyzed separately. Previous studies have also used these items individually to measure cancer fatalism [31,32].

Cancer information seeking was measured with a single item asking whether respondents had ever looked for information about cancer from any source, with responses recorded as “yes” or “no.” Interpersonal discussion about health was also assessed with a single item asking whether participants had talked with friends or family members about their health. This variable, reflecting informal health-related communication within personal social networks, was similarly recorded as “yes” or “no”.

#### Covariates.

All models adjusted for sociodemographic and cancer-related factors known to be associated with depressive symptoms and the IVs. Sociodemographic variables included age, sex assigned at birth, education, household income and health insurance coverage, factors consistently associated with mental health disparities and health information engagement [32–36]. Personal and family history of cancer were also included, given that direct or familial experiences with cancer can affect emotional responses, shape beliefs about cancer risk, and influence information-seeking behaviors [4,37].

### Statistical analysis

Descriptive statistics were computed for all study variables and stratified by depression screening status. Categorical variables are presented as unweighted frequencies and weighted percentages, while continuous variables are summarized using weighted medians and interquartile ranges (IQRs). Bivariate associations between depression status and each independent or covariate variable were examined using survey-weighted chi-square tests (with Rao–Scott adjustment) for categorical variables and design-based Kruskal–Wallis tests for continuous variables, accounting for the complex sampling design of HINTS 7.

Primary analyses were conducted using complete-case data, excluding respondents with missing values on any variable included in the corresponding model. Separate multivariable logistic regression models were fitted for each hypothesized association, adjusting for the covariates. All analyses incorporated person-level survey weights and jackknife replicate weights to account for the complex sampling design and produce nationally representative estimates. Adjusted odds ratios (ORs) and 95% confidence intervals (CIs) are reported.

In a sensitivity analysis, we examined whether these associations differed among individuals without a personal history of cancer. Respondents who had ever been diagnosed with cancer were excluded to ensure that results were not driven by cancer survivors, who may differ systematically in psychological distress or health information engagement. The same survey-weighted logistic regression models were re-estimated in this restricted subsample, maintaining identical model specifications.

We also explored the potential impact of missing data on the study findings by implementing multiple imputation. Approximately 12.7% of respondents (unweighted *n* = 867 out of 6,826) had at least one missing value. Multiple imputations were conducted using the *mice* package in R, following the fully conditional specification approach under the assumption that data were missing at random (MAR) [38]. Binary variables like personal cancer history, cancer beliefs, and cancer information seeking were imputed using logistic regression, while variables with more than two levels (sex, education, family history of cancer, income) were imputed using polytomous logistic regression. Age, as a continuous variable, was imputed using predictive mean matching. All variables, including the outcome, were included as predictors in the imputation models to preserve relationships among variables, as recommended by Austin et al. [39]. Following recommendations by White et al. [40], we generated 13 imputed datasets, corresponding to the approximate proportion of respondents with missing data. Each imputed dataset was analyzed using the same complete-case specifications, and resulting estimates were combined using Rubin’s Rules [41] to produce pooled ORs with corresponding 95% CIs.

All analyses were conducted using R version 4.5.1, and statistical significance was assessed at *p* < 0.05.

## Results

### Sample characteristics

Table 1 summarizes weighted characteristics of the analytic sample (unweighted *N* = 6,826; weighted *N* = 248.3 million U.S. adults). Overall, 15.5% of respondents (weighted *N* = 38.6 million) screened positive for depression based on the PHQ-2. The median age of the total (weighted) sample was 49 years (IQR: 34–63), with those screening positive being significantly younger than those who screened negative (median = 40 vs. 50 years, *p* < 0.001). Educational attainment and income differed significantly by depression status. Only 24% of respondents who screened positive were college graduates, compared with 36% of those who screened negative (*p* < 0.001). Similarly, 31% of those with positive depression screening reported annual household incomes below $20,000, compared with 12% of those with negative screening (*p* < 0.001). Health insurance coverage followed a similar pattern: 85% of respondents who screened positive were insured, compared to 93% of those who screened negative (*p* < 0.001). Depression prevalence did not differ by personal cancer history (*p* = 0.80) but varied by family history: 59% of respondents who screened positive reported having a family history of cancer, compared with 66% of those who screened negative (*p* = 0.041). Although cancer information seeking did not differ significantly by depression screening status (48% vs. 52%, *p* = 0.20), differences were observed in interpersonal health communication: fewer respondents who screened positive reported discussing health with family or friends (74% vs. 82%, *p* = 0.001). Finally, a higher proportion of respondents who agreed with fatalistic cancer beliefs screened positive for depression.

**Table 1:** Descriptive characteristics of respondents by depression screening outcome^a^^.^

		Depression screening status		p-value^b^
Characteristics	Overall N = 6,826	Positive N = 1,047	Negative N = 5,779	
**Age, yrs – Median (Q1, Q3)**	49 (34, 63)	40 (29, 54)	50 (36, 64)	<0.001
Missing	266	51	215	
**Sex assigned at birth**				0.200
Male	2,608 (51%)	345 (47%)	2,263 (52%)	
Female	3,942 (48%)	645 (52%)	3,297 (48%)	
Unknown	44 (1%)	11 (1%)	33 (1%)	
Missing	232	46	186	
**Educational attainment**				<0.001
College graduate or more	3,161 (34%)	341 (24%)	2,820 (36%)	
High school or less	1,511 (27%)	330 (39%)	1,181 (25%)	
Post high school or some college	1,914 (39%)	326 (38%)	1,588 (39%)	
Missing	240	50	190	
**Annual household income**				<0.001
less than $20,000	1,089 (15%)	314 (31%)	775 (12%)	
$20,000 to less than $50,000	1,559 (23%)	288 (30%)	1,271 (22%)	
$50,000 to less than $100,000	1,797 (28%)	242 (25%)	1,555 (28%)	
$100,000 or more	1,847 (34%)	126 (15%)	1,721 (38%)	
Missing	534	77	457	
**Any health insurance coverage**	6,173 (91%)	879 (85%)	5,294 (93%)	<0.001
Missing	34	8	26	
**Ever had cancer**	1,049 (10%)	149 (10%)	900 (10%)	0.800
Missing	193	40	153	
**Family history of cancer**				0.041
Yes	4,367 (65%)	605 (59%)	3,762 (66%)	
No	1,392 (22%)	230 (24%)	1,162 (22%)	
Not sure	719 (13%)	146 (17%)	573 (12%)	
Missing	348	66	282	
**Sought cancer information**	3,538 (51%)	480 (48%)	3,058 (52%)	0.200
Missing	2	0	2	
**Talk to family or friends about health**	5,604 (81%)	770 (74%)	4,834 (82%)	0.001
Missing	33	8	25	
**Everything causes cancer**	4,468 (69%)	753 (78%)	3,715 (68%)	<0.001
Missing	256	55	201	
**Cancer prevention is not possible**	1,995 (31%)	455 (45%)	1,540 (28%)	<0.001
Missing	279	60	219	
**Cancer automatically means death**	3,842 (62%)	712 (75%)	3,130 (60%)	<0.001
Missing	264	57	207	

^a^Unweighted frequencies shown; percentages are weighted and may not sum to 100 due to rounding.

^b^Survey-weighted chi-squared tests for categorical variables and design-based Kruskal–Wallis tests for continuous variable (age).

**NOTE**: Weighted percentages and median are based on HINTS 7 survey weights. The total weighted sample size is approximately N = 248,274,572, of which 38,603,460 (15.5%) screened positive for depression based on the PHQ-2 scale.

### Adjusted associations between cancer beliefs, information behaviors, and depression screening

Across all three belief items, fatalistic views of cancer were linked to higher odds of depression (Table 2, Overall sample). The odds of screening positive for depression among respondents who agreed that cancer prevention is not possible were 1.69 times the odds among those who disagreed (OR = 1.69, 95% CI: 1.25–2.28). Similarly, respondents who agreed that everything causes cancer had 1.86 times the odds of screening positive for depression compared with those who disagreed (OR = 1.86, 95% CI: 1.39–2.48), and those who endorsed that cancer automatically means death had 1.75 times the odds of screening positive compared with those who disagreed (OR = 1.75, 95% CI: 1.31–2.34). In contrast, cancer information seeking was not significantly associated with depression screening (OR = 1.12, 95% CI: 0.83–1.51). Likewise, the odds of screening positive for depression did not differ significantly between respondents who talked with family or friends about health and those who did not (OR = 0.90, 95% CI: 0.62–1.30).

**Table 2:** Survey-weighted logistic regression models of associations between fatalistic beliefs, information seeking, interpersonal communication, and depression screening among U.S. adults and non-cancer adults.

	Overall sample (N = 5,959)		Non-cancer adults^a^ (N = 5,026)	
	OR	95% CI	OR	95% CI
**(1) Fatalistic cancer beliefs**				
Cancer prevention is not possible	1.69**	(1.25, 2.28)	1.71**	(1.25, 2.34)
Everything causes cancer	1.86***	(1.39, 2.48)	1.88***	(1.36, 2.61)
Cancer automatically means death	1.75***	(1.31, 2.34)	1.77***	(1.28, 2.43)
**(2) Information seeking**				
Seek cancer information	1.12	(0.83, 1.51)	1.11	(0.82, 1.50)
**(3) Interpersonal communication**				
Talk to family/friends about health	0.90	(0.62, 1.30)	0.90	(0.62, 1.32)
**(4) Interaction model** ^b^				
Cancer prevention is not possible	1.33	(0.61, 2.88)	1.32	(0.59, 2.98)
Everything causes cancer	0.95	(0.50, 1.80)	0.96	(0.47, 1.93)
Cancer automatically means death	0.79	(0.38, 1.66)	0.77	(0.35, 1.67)

**Note**. Each row represents a separate survey-weighted logistic regression model examining the association between the listed variable and depression screening status (PHQ-2 ≥ 3). Models incorporated person-level weights and jackknife replicate weights to account for the complex HINTS 7 sampling design. All models adjusted for age, sex assigned at birth, education, income, health insurance coverage, personal cancer history, and family cancer history. Models restricted to non-cancer respondents excluded the personal cancer history variable. For cancer fatalism variables, the reference group is respondents who disagreed with the statement. For cancer information seeking and interpersonal health communication, the reference group is respondents who answered “No.” *p < 0.05, **p < 0.01, ***p < 0.001.

^a^Represents those who responded “No” to the question “Have you ever been diagnosed as having cancer?”

^b^Each interaction model included a cross-product term between each cancer fatalism variable and interpersonal health communication. Reported estimates represent the effects of endorsing fatalistic beliefs and engaging in interpersonal communication relative to their respective reference groups.

In the moderation analyses (Table 2, Interaction model), none of the interaction terms were statistically significant. The interaction between talking with family or friends and the belief that cancer prevention is not possible was positive but not significant (OR = 1.33, 95% CI: 0.61–2.88), while the interactions for “everything causes cancer” (OR = 0.95, 95% CI: 0.50–1.80) and “cancer automatically means death” (OR = 0.79, 95% CI: 0.38–1.66) were negative but not significant.

Sensitivity analyses using multiple imputation and limiting the analyses to only non-cancer adults showed consistent results with the complete-case models, and the overall sample. Associations between fatalistic beliefs and depression remained positive and statistically significant, while the direction and magnitude of estimates for information seeking and interpersonal communication were largely unchanged (see S1 Table).

## Discussion

This study examined how people’s beliefs about cancer, their information-seeking behaviors, and conversations with family or friends about health relate to depressive symptoms, using nationally representative data from HINTS 7. Consistent with past research, those who held fatalistic cancer beliefs, particularly the perceptions that everything causes cancer, that prevention is not possible, or that cancer automatically means death, had higher odds of screening positive for depression, controlling for socio-demographics and cancer history. In contrast, neither cancer information seeking nor talking with family or friends about health showed a significant association with depression, though the latter exhibited a suggestive (non-significant) buffering pattern, slightly weakening the association between some fatalism items and depression screening. Sensitivity analyses using multiple imputation for missing data and analyses restricted to respondents without a personal cancer history yielded substantively identical results, reinforcing the stability of these findings.

The significant positive association between fatalistic cancer beliefs and depressive symptoms shows the emotional toll of perceiving cancer as uncontrollable or inevitable. Fatalistic thinking captures elements that overlap conceptually with core depressive cognitions [9,10,42,43]; this plausibly links fatalistic outlooks to sustained negative emotion, withdrawal, and reduced problem-focused coping. Such beliefs not only discourage preventive actions but also erode perceived control and optimism, limiting people’s ability to cope adaptively. Empirically, our findings extend prior literature that ties fatalism to lower screening and prevention [7,8] by demonstrating that these cognitions also map onto mental health in the broader population, including those without a cancer diagnosis, reinforcing the idea that cancer beliefs are simultaneously behavioral and emotional determinants. Similar patterns appear in other areas of health research, where fatalistic determinism are linked to higher depression and lower resilience [11,44].

Theoretically, these results align with the C-SHIP model, which emphasizes how cognitive and emotional systems interact to shape responses to health threats. Fatalistic beliefs operate as maladaptive cognitive schemas that bias attention and appraisal toward threat and helplessness rather than solutions. Repeatedly interpreting cancer information through this lens can deplete coping resources, undermine perceived control, and reinforce hopeless affect. This parallels Beck’s cognitive theory of depression [45], which describes how enduring negative schemas sustain depression cognition. In this sense, fatalistic cancer beliefs represent a cognitive, emotional pattern that traps individuals in cycles of fear and resignation, increasing vulnerability to depression.

Given the emotional burden of fatalistic beliefs, seeking information may represent a potential coping strategy, yet its impact on depressive symptoms is complex and not always beneficial. Results indicated that the association between information seeking, and depression screening was not statistically significant, though the positive direction of the OR suggests a subtle pattern worth considering. Information seeking is typically portrayed as an empowering behavior that enhances knowledge and self-efficacy [20], yet its emotional consequences may vary across individuals and contexts. According to UMT [17–19], people seek information not merely to gain facts but to regulate uncertainty, an effort that can either reduce or heighten distress depending on their perceptions of control and the nature of the information encountered [25,46]. The modern information environment, however, is noisy and emotionally charged; people are bombarded with alarming headlines, conflicting risk statistics, and fear-evoking narratives that can amplify worry rather than reassurance [47]. For individuals who already hold fatalistic or anxious views about cancer, this constant exposure may intensify emotional fatigue, offsetting the potential benefits of seeking information. The nonsignificant finding could therefore reflect these competing processes: while some use information seeking to regain a sense of control, others experience cognitive overload, producing an overall null effect at the population level. Future research using more detailed measures of information quality, credibility, and emotional tone could help clarify when seeking health information helps individuals versus when it overwhelms them.

A similar story may explain the lack of a significant main effect for interpersonal communication. Social interactions with family and friends are commonly viewed as a potential buffer against emotional distress [48], yet simply reporting that one talks about health may be insufficient; the content and tone of those conversations likely matter more. Supportive discussions can help people process emotions and find reassurance, but conversations filled with fear or misinformation can have the opposite effect [49,50]. Our moderation analyses add nuance to these dynamics. The pattern of results (showing ORs slightly below 1 for some fatalism items when interpersonal talk was included) suggest that interpersonal communication may buffer emotional distress. In other words, discussing health with trusted others may weaken the distressing effect of fatalism, but our study lacked sufficient precision to confirm it. This buffering effect may not have been detected statistically, likely because the present data captured only whether people discussed health, not how often or how constructively they did so. Future studies should explore this potential moderation using richer measures of conversation frequency and quality, continuous fatalism scales, and larger samples designed for adequate interaction power.

Taken together, these findings have important implications for health communication practice. Public health messages should not only share information but also attend to how people interpret and feel about that information. Messages that focus too much on risk or uncertainty without emphasizing prevention or recovery, can unintentionally reinforce fatalistic thinking and feelings of overload or distress [51]. More effective campaigns should balance honesty about risk with clear, hopeful, and actionable guidance. Including stories of survival, progress in treatment, and examples of control may help people feel capable rather than doomed, as narrative communication has been shown to reduce resistance and emotional barriers to cancer prevention [52]. Finally, communicators must consider inequities in literacy and access. People with limited resources or lower health literacy may experience complex cancer messages as confusing or threatening, necessitating a shift toward emotionally sensitive and accessible design for all audiences [53–55].

Despite the study’s contributions, it has some limitations. Because the data is cross-sectional, it is not possible to determine cause and effect: depression might also lead people to develop more fatalistic beliefs, or both could reinforce each other. Also, self-reported measures may include recall bias or social desirability effects, and the PHQ-2 identifies depressive symptoms but does not diagnose clinical depression. Fatalism items captured only a certain facet of fatalistic belief and do not include factors such as medical distrust or perceptions of randomness. Moreover, it is also possible that unmeasured factors like overall social support, media exposure, or baseline anxiety, contributed to the observed relationships. Future research should use longitudinal or experimental designs, more detailed measures of fatalism and communication quality, and qualitative methods to better understand how people emotionally navigate conflicting cancer information.

In conclusion, this study shows that how people think and talk about cancer can affect how they feel. Fatalistic beliefs were consistently associated with depression, while interpersonal communication showed hints of being protective. These findings highlight that accurate information alone is not sufficient; health messages must also foster hope, confidence, and emotional safety. When communication acknowledges fear and also offers clarity and control, it can help people feel informed without feeling defeated.

## Supporting information

S1 Table Results from multiple imputation analyses (13 imputations). Survey-weighted logistic regression estimates of the associations between fatalistic beliefs, health information seeking, discussions about health with family or friends, and depression are presented as adjusted ORs with 95% CIs. Estimates from the 13 imputed datasets were pooled using Rubin’s rules.
(DOCX)
