# Supplementary material for: Helpful or harmful? How cancer beliefs and information seeking relate to depression in U.S. adults
Source: PLoS One. 2026 Apr 2;21(4):e0346262. doi: 10.1371/journal.pone.0346262 (PMC13046162; doi:10.1371/journal.pone.0346262)
Supplement: S1 Table — Survey-weighted logistic regression estimates of the associations between fatalistic beliefs, health information seeking, discussions about health with family or friends, and depression are presented as adjusted ORs with 95% CIs. Estimates from the 13 imputed datasets were pooled using Rubin’s rules. (DOCX) [file pone.0346262.s001.docx]

**S1 Table. Survey-weighted logistic regression models of associations between fatalistic beliefs, information seeking, interpersonal communication, and depression screening after multiple imputation.**

|  | **Overall sample**  **(N = 6,826)** | |  | **Non-cancer adults**  **–** | |
| --- | --- | --- | --- | --- | --- |
|  | **OR** | **95% CI** |  | **OR** | **95% CI** |
| **(1) Cancer fatalism beliefs** |  |  |  |  |  |
| Prevention not possible | 1.78*** | (1.36, 2.32) |  | 1.78*** | (1.34, 2.39) |
| Everything causes cancer | 1.80*** | (1.38, 2.35) |  | 1.77*** | (1.30, 2.41) |
| Cancer automatically means death | 1.79*** | (1.38, 2.33) |  | 1.77*** | (1.32, 2.38) |
| **(2) Information seeking** |  |  |  |  |  |
| Seek cancer information | 1.06 | (0.80, 1.42) |  | 1.10 | (0.83, 1.47) |
| **(3) Interpersonal communication** |  |  |  |  |  |
| Talk to family/friends about health | 0.83 | (0.59, 1.17) |  | 0.86 | (0.60, 1.22) |
| **(4) Interaction model** |  |  |  |  |  |
| Prevention not possible | 1.34 | (0.67, 2.70) |  | 1.40 | (0.67, 2.92) |
| Everything causes cancer | 1.01 | (0.58, 1.78) |  | 1.03 | (0.56, 1.88) |
| Cancer automatically means death | 0.87 | (0.46, 1.66) |  | 0.83 | (0.42, 1.64) |

**Note**. Models are survey-weighted logistic regressions adjusted for age, sex assigned at birth, education, income, health insurance coverage, personal cancer history, and family cancer history. Results are pooled across 13 multiply imputed datasets.

Each row represents a separate survey-weighted logistic regression model examining the association between the listed variable and depression screening status (PHQ-2 ≥ 3). Models incorporated person-level weights and jackknife replicate weights to account for the complex HINTS 7 sampling design.

****p*<0.001
